# Individual and social determinants of multiple chronic disease behavioral risk factors among youth

**DOI:** 10.1186/1471-2458-12-224

**Published:** 2012-03-22

**Authors:** Arsham Alamian, Gilles Paradis

**Affiliations:** 1 Department of Biostatistics and Epidemiology, College of Public Health, East Tennessee State University, Johnson City, Tennessee, USA; 2 Department of Epidemiology, Biostatistics and Occupational Health, Faculty of Medicine, McGill University, Montreal, Quebec, Canada; 3 Research Institute of the McGill University Health Centre, Montreal, Quebec, Canada

## Abstract

**Background:**

Behavioral risk factors are known to co-occur among youth, and to increase risks of chronic diseases morbidity and mortality later in life. However, little is known about determinants of multiple chronic disease behavioral risk factors, particularly among youth. Previous studies have been cross-sectional and carried out without a sound theoretical framework.

**Methods:**

Using longitudinal data (n = 1135) from Cycle 4 (2000-2001), Cycle 5 (2002-2003) and Cycle 6 (2004-2005) of the National Longitudinal Survey of Children and Youth, a nationally representative sample of Canadian children who are followed biennially, the present study examines the influence of a set of conceptually-related individual/social distal variables (variables situated at an intermediate distance from behaviors), and individual/social ultimate variables (variables situated at an utmost distance from behaviors) on the rate of occurrence of multiple behavioral risk factors (physical inactivity, sedentary behavior, tobacco smoking, alcohol drinking, and high body mass index) in a sample of children aged 10-11 years at baseline. Multiple behavioral risk factors were assessed using a multiple risk factor score. All statistical analyses were performed using SAS, version 9.1, and SUDAAN, version 9.01.

**Results:**

Multivariate longitudinal Poisson models showed that social distal variables including parental/peer smoking and peer drinking (Log-likelihood ratio (LLR) = 187.86, degrees of freedom (DF) = 8, *p *< .001), as well as individual distal variables including low self-esteem (LLR = 76.94, DF = 4, *p *< .001) increased the rate of occurrence of multiple behavioral risk factors. Individual ultimate variables including age, sex, and anxiety (LLR = 9.34, DF = 3, *p *< .05), as well as social ultimate variables including family socioeconomic status, and family structure (LLR = 10.93, DF = 5, *p *= .05) contributed minimally to the rate of co-occurrence of behavioral risk factors.

**Conclusions:**

The results suggest targeting individual/social distal variables in prevention programs of multiple chronic disease behavioral risk factors among youth.

## Background

Chronic (long-lasting) diseases including heart disease, stroke, cancer and diabetes are by far the leading causes of death worldwide [1]. Behavioral risk factors including tobacco smoking, alcohol drinking, physical inactivity, sedentary behavior, and obesity are major determinants of adult chronic diseases morbidity and mortality [2,3]. For instance, nearly 80% of incident cases of cardiovascular disease and type 2 diabetes are attributable to physical inactivity, tobacco smoking and unhealthy diet alone [1]. About 35% of all cancers are also preventable by reducing or avoiding exposure to risk factors such as tobacco use, physical inactivity, poor diet, alcohol use or being overweight or obese [4].

Chronic disease behavioral risk factors originate in childhood and adolescence [5-9], and cause significant negative health and social consequences throughout the life course [2,10,11]. In particular, physically inactivity has been linked to an unfavorable cardiovascular disease risk profile including obesity [12], insulin resistance [10], and high blood pressure [13]. Sedentary behavior has been associated with being overweight as it involves a decrease in energy expenditure and an increase in energy intake through consumption of high-fat and low-nutrient foods [14]. Smoking at a young age has been associated with emotional and psychological problems, engaging in risky behaviors such as violence and sexual activity, and an increased risk for lung cancer later in life [15,16]. Underage alcohol drinking has been suggested to increase rates of suicide and homicide, and even death from alcohol poisoning [17]. Lastly, obesity during childhood has been linked to increased risk of dyslipidemia, hyperinsulinemia, hypertension, and a number of psychosocial problems [18,19].

In addition to the burden of disease attributed to single chronic behavioral risk factors, a growing body of evidence also suggests that behavioral risk factors (including physical inactivity, sedentary behavior, smoking, alcohol use, and obesity) co-occur among youth [20-23], and that their combinations yield greater risks for chronic diseases than the sum of their individual independent effects [24]. Although much is known about single behavioral risk factors and their determinants, less is known about potential determinants of multiple behavioral risk factors, particularly among youth. Previous studies of multiple behavioral risk factors for chronic diseases have been cross-sectional; these studies have identified a limited number of individual characteristics, such as being female [22,25], older age [25-27], depression [26,28] and low self-esteem [27], as well as social characteristics, including living in a lone-parent family [26,27], low parental education [27] and parental unhealthy lifestyles [25,27] as correlates of multiple behavioral risk factors among youth. While these findings are important, there is a need for longitudinal studies to obtain more conclusive evidence for planning cost-effective interventions.

Identifying factors that contribute to the co-occurrence of health behaviors should be based on a theory applicable to multiple behaviors [29]. However, previous studies of multiple behavioral risk factors have not consistently used a sound theoretical framework [22,25,28,30]. In addition, several theories of health behavior, including the Health Belief Model [31], the Theory of Reasoned Action [32] and the Theory of Planned Behavior [33] are considered behavior-specific, because these theories suggest that each behavior has its own set of determinants, commonly referred to as proximal factors (i.e., the most immediate determinants of specific behaviors) [34,35]; these include attitudinal, social normative beliefs, self-efficacy and decisional/intentional factors [29].

Other prominent theories including the Social Learning Theory [36], the Problem Behavior Theory [37], the Bronfenbrenner's Ecological Systems Theory [38], and the Theory of Triadic Influence [39] address more distal determinants of behaviors such as self-esteem, social bonding with others as well as characteristics of the social environment. However, of all integrative theories, the Theory of Triadic Influence seems to be the most comprehensive as it proposes a framework for mapping out the relationships between determinants of different types (including individual and social characteristics) and the occurrence of both single and multiple behaviors [34,39]. According to the Theory of Triadic Influence, individual and social factors influence health behaviors through 3 tiers of constructs, represented by several proximal, distal and ultimate variables. Flay and Petraitis [39] argue that as opposed to proximal variables which are behavior-specific, distal and ultimate variables are likely to have more generalizable effects and thus, they are thought to be predictive of multiple behaviors [35]. In particular, ultimate variables are the most general set of principles that transcend specific behaviors, and they comprise factors considered almost unchangeable such as inherited dispositions (e.g., sex, age), or factors that are difficult to change such as personality traits (e.g., anxiety) and characteristics of the social environment (e.g. family socioeconomic status) [39]. Hence, ultimate variables are considered to be furthest from behavior(s), in terms of distance, and believed to be not specific to a single behavior. As a result, ultimate variables are hypothesized to strongly influence multiple behaviors. Distal variables are more immediate determinants of behavior(s) (compared to ultimate variables), and they comprise factors considered easier to modify, such as one's general knowledge, social relations and sense of self [39]. Distal variables are also hypothesized to influence multiple behaviors, but to a lesser degree compared to ultimate variables, since they are closer to behavior(s) (i.e., they are assumed to exert less generalized effects across behaviors).

To our knowledge, no study has yet investigated the longitudinal relation between a large set of distal and ultimate variables and the occurrence of multiple behavioral risk factors for chronic diseases among youth. The present study is therefore guided by the Theory of Triadic Influence and uses longitudinal data to examine the influence of selected individual/social distal and ultimate variables on the rate of occurrence of multiple behavioral risk factors in a representative sample of Canadian youth. We hypothesized that both distal and ultimate variables would influence the rate of co-occurrence of chronic disease behavioral risk factors among youth. However, ultimate determinants would be expected to exert a stronger influence on the rate of occurrence of multiple chronic disease behavioral risk factors, compared to distal determinants, due to their potentially broader effects.

## Methods

### Study population

The National Longitudinal Survey of Children and Youth (NLSCY) is a large representative survey of Canadian children and adolescents that follows their development and well-being from birth to adulthood. The NLSCY uses a stratified, multistage probability sample design with data collection occurring at two-year intervals [40]. The present analysis was based on a weighted longitudinal sample of Canadian children aged 10-11 years in Cycle 4 (2000-2001), 12-13 years in Cycle 5 (2002-2003) and 14-15 years in Cycle 6 (2004-2005) of the NLSCY. Of 2081 children aged 10-11 years in Cycle 4, 1838 (88.3%) responded to Cycle 5. Of these 1838 youth, 1649 (79.2% of the original sample) responded to Cycle 6. Of these 1649 youth, analyses were based on 1135 youth (68.9%) with complete data on lifestyle variables and covariates. Table 1 presents the baseline characteristics of children included in the study population and of those lost to follow-up or excluded because of incomplete data. This study received approval from the Ethics Committee on Research on Human Subjects of the Faculty of Medicine of the University of Montreal.

**Table 1:** Comparison of baseline characteristics of children in the study cohort and of subjects lost to follow-up or excluded because of incomplete data, National Longitudinal Survey of Children and Youth, 2000-2005

	Study cohort, %^a^	Subjects lost, %^a^	*p *value^b^
	
	(n = 1135)	(n = 946)	
**Individual characteristics**			

**Ultimate**			

Sex			.03

Female	51	46	

Age, years			.26

10	50	53	

11	50	47	

Anxiety, mean (SE)^c^	3.4 (0.1)	3.7 (0.1)	.02

**Distal**			

Self-esteem, mean (SE)^d^	13.7 (0.1)	13.3 (0.1)	<.001

Academic performance			.56

Poor/very poor	2	1	

Average	18	19	

Well	46	46	

Very well	34	34	

**Social characteristics**			

**Ultimate**			

Family structure			.005

2 parents	84	79	

1 parent	16	21	

PMK Education			<.001

Low (< 12 years of school)	19	28	

High (≥12 years of school)	81	73	

Annual household income, CAN $			<.001

< 30,000	15	21	

30,000-59,999	31	40	

60,000-89,999	31	23	

≥ 90,000	23	16	

**Distal**			

PMK smoking status			.03

Tobacco smoker	26	30	

PMK drinking status			.61

Alcohol drinker	28	27	

Parent-child relationship, mean (SE)^e^	22.9 (0.2)	22.3 (0.2)	.005

Peer smoking			.94

No peers	95	96	

A few peers	4	3	

Most/all peers	1	1	

Peer drinking			.05

No peers	97	95	

A few peers	2	4	

Most/all peers	1	1	

Peer-child relationship, mean (SE)^f^	12.8 (0.1)	12.8 (0.1)	.86

**Lifestyle risk factors**			

Physical inactivity^g^	50	54	.09

Sedentary behavior^h^	42	46	.11

Ever smoking^i^	6	7	.60

Ever drinking^j^	6	10	.005

High body mass index^k^	23	29	.004

CAN = Canadian; PMK = person most knowledgeable; SE = standard error.

^a ^Weighted percentage expressed in terms of the proportion of Canadian children aged 10-11 years in Cycle 4 and followed biennially until Cycle 6 of the National Longitudinal Survey of Children and Youth.

^b ^*p *value from a chi-squared test or *t *test.

^c ^Anxiety was assessed using a global score ranging from 0 to 14, with higher scores indicating the presence of greater anxiety.

^d ^Self-esteem was assessed using a global score ranging from 0 to 16, with higher scores indicating positive self-esteem.

^e ^The parent-child relationship was assessed using a global score ranging from 0 to 28, with higher scores indicating a better relationship between parents and child.

^f ^Peer-child relationships were assessed using a global score ranging from 0 to 16, with higher scores indicating a better relationship between the child and his/her peers.

^g ^Engaging in organized/unorganized physical activities fewer than 4 times per week.

^h ^Watching television or videos for more than 2 hours per day.

^i ^Ever smoking a cigarette, even a few puffs.

^j ^Ever having a standard drink of alcohol.

^k ^Being overweight/obese, as defined by cutoff points of Cole and colleagues [54].

### Data collection

The person most knowledgeable (PMK) about the child, most often the mother, completed a parent questionnaire and a child questionnaire. The parent questionnaire gathered information on family socioeconomic status and PMK adverse health behaviors, while the child questionnaire was used to obtain the child's height and weight (for children below age 12 years). Specifically, the PMK was asked to indicate the child's height in meters and centimetres, and to report the child's weight in kilograms and grams. Adolescents aged 12 years or more self-reported their height and weight. Information regarding youth behaviors and social relations was assessed through age-specific self-administered questionnaires for children aged 10 years or more.

### Measures

#### Risk factors

Physical inactivity was measured in Cycles 4, 5 and 6 using 2 closed questions adapted from the World Health Organization Health Behavior in School-aged Children (HBSC) survey: 1) "During the past 12 months, how often have you played sports or done physical activities without a coach or an instructor (biking, skateboarding, etc.)?"; 2) "During the past 12 months, how often have you played sports with a coach or an instructor, other than gym class (swimming lessons, baseball, hockey, etc.)?" [41]. Response choices included "never", "less than once a week", "1 to 3 times a week" and "4 or more times a week". Because the Canadian *Physical activity Guides for Children and Youth *[42] recommend daily participation in physical activities, we defined physical inactivity as engaging in organized/unorganized activities fewer than 4 times per week. The physical activity questions have been validated by means of the Multistage Fitness Test [43], and have been shown to have acceptable validity. The intra-class correlation coefficient for the reliability of this measure was 0.74, in the targeted age groups [44].

Sedentary behavior was measured in Cycles 4, 5 and 6 using a closed question from the HBSC survey: "On average, about how many hours a day do you watch television or videos?" [41]. Because the American Academy of Pediatrics guidelines recommend limiting screen viewing to 2 hours per day or less [45], we defined sedentary behavior as watching television or videos for more than 2 hours per day. The sedentary behavior measure has been validated using a 7-day television viewing diary. Spearman correlation coefficients ranged from 0.36 to 0.54 [46]. Test-retest intra-class correlation scores for the reliability of this measure ranged from 0.76 to 0.81 [41,46].

Cigarette smoking was assessed using a closed question adapted from the HBSC survey asking youth about their past experience with tobacco smoking [41]. Previous research has indicated that any cigarette use places the child at greater risk for subsequent use and children who begin smoking at an early age are more likely to develop severe nicotine addiction than those who start later [47,48]. Thus, we used Health Canada's definition of ever smoking, that is, having ever tried a cigarette, even a few puffs [49], in Cycles 4, 5 and 6.

Alcohol drinking was assessed using two closed questions inquiring about past experience with alcohol consumption [41,50]. Longitudinal studies have shown that children who start drinking (more than just a few sips) as early as 11 years of age are at increased risk of becoming problem and heavy drinkers later in life [51,52]. Thus, we defined alcohol drinking as ever drinking, that is ever having had at least 1 alcoholic drink, as suggested by others [53], in Cycles 4, 5 and 6.

High body mass index (weight (kg)/height (m)^2^) was defined as overweight or obese, in all three cycles, according to Cole and colleagues' [54] international age- and sex-specific body mass index cutoffs for children and adolescents, corresponding to body mass indices of 25 and 30, respectively, at age 18 years.

#### Independent variables

**Figure 1:**
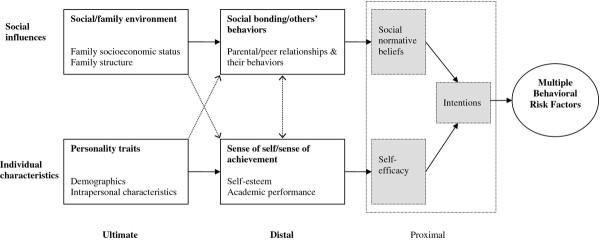
**Conceptual framework of the influence of ultimate and distal variables on multiple behavioral risk factors (Adapted from the Theory of Triadic Influence **[39]). Proximal variables are only presented in this framework to suggest a pathway through which individual/social distal and ultimate variables might influence multiple behavioral risk factors. Dotted arrows represent possible interstream pathways between the ultimate and the distal variables.

The independent variables were selected on the basis of factors previously identified as correlates of several lifestyle risk factors in the literature and comprised four blocks of variables: individual ultimate, individual distal, social ultimate and social distal variables, as per our conceptual framework based on the Theory of Triadic Influence (Figure 1).

*Individual ultimate *variables referred to demographic and personality factors such as sex, age at baseline (10-11 years in Cycle 4), and anxiety. Anxiety was assessed, in Cycles 4 through 6, using 7 questions from the Ontario Child Health Study assessing degree of nervousness, anxiety and depression [55]. Based on the responses, a global score ranging from 0 to 14 was calculated, with higher scores indicating the presence of greater anxiety. This measure has been validated through factor analyses and has been shown to have good construct validity. Its reliability was also satisfactory (Cronbach's α = 0.76) in the NLSCY [50].

*Individual distal *variables referred to child's sense of self and achievement such as self-esteem and academic performance. Self-esteem was measured, in Cycles 4 through 6, using 4 items from the General Self-Scale of the Marsh Self-Description Questionnaire [56]. A global score ranging from 0 to 16 was computed, with higher scores indicating positive self-esteem. This measure has been shown to have high convergent validity (factor intercorrelation = 0.76) [57]. Its reliability was also satisfactory (Cronbach's α = 0.73) in the NLSCY [50]. Academic performance was assessed, in Cycles 4 through 6, using a closed question: "How well do you think you are doing in your school work?" [50]. Response choices included: "very well", "well", "average", "poor" and "very poor". In the analyses, the response categories "poor" and "very poor" were combined to ensure adequate cell sizes.

*Social ultimate *variables referred to characteristics of the child's immediate social environment such as family structure (2 parents, 1 parent); PMK education defined as low education (< 12 years of schooling) and high education (12 years of schooling or more) [58,59]; and total annual household income (< Can$30,000, Can$30,000-59,999, Can$60,000-89,999 or ≥ Can$90,000) [60], assessed in all cycles.

*Social distal *variables pertained to child's social relations with others as well as behaviors of influential role models. PMK smoking was defined as smoking "daily" or "occasionally", in all cycles [50]. PMK drinking was defined as consuming alcohol at least once a week or more, in all cycles [50,61]. The parent-child relationship was assessed, in Cycles 4 through 6, using 7 questions from the Western Australia Child Health Survey evaluating the child's perception of the parents' degree of attention, appreciation and affection [50]. A global score ranging from 0 to 28 was computed, with higher scores indicating better parent-child relationships. The reliability of this scale was excellent (Cronbach's α = 0.88) in the NLSCY [50]. Peer smoking and peer drinking were assessed, in Cycles 4 through 6, using 2 closed questions: "How many of your close friends smoke cigarettes"? and "How many of your close friends drink alcohol?" [50]. Response choices included "none", "a few", "most" and "all". In the analyses, response categories "most" and "all" were combined to ensure adequate cell sizes. Peer-child relationships were assessed, in Cycles 4 through 6, using 4 items from the Ontario Child Health Study evaluating how well the child feels he/she gets along with his/her peers [55]. A global score ranging from 0 to 16 was computed, with higher scores indicating better relationships with peers. The reliability of this scale was satisfactory (Cronbach's α = 0.78) in the NLSCY [50].

### Statistical analyses

Baseline characteristics of the study cohort were described using the chi-squared test and t-test. The prevalence and 95% confidence intervals (CIs) of single and multiple behavioral risk factors by sex were estimated using sampling and bootstrap weights [40]. Single behavioral risk factors were coded as binary variables (yes = 1, no = 0). A multiple risk factor score ranging from 0 to 5 (0 = no risk factors, 5 = all 5 risk factors) was then created by summing individual risk factor scores [25]. Sex-specific trends in the percentage of single and multiple behavioral risk factors were examined using polynomial trend tests [62]. We used longitudinal Poisson regression, within a generalized estimating equations (GEE) framework, to assess the longitudinal associations between selected individual/social distal and ultimate variables and the multiple risk factor score. GEE models account for non-independence of repeated observations and provide robust parameter and standard error estimates [63]. In addition, the longitudinal Poisson regression models provided direct estimates of rate ratios for the associations between selected covariates and the multiple risk factor score along the entire follow-up period [64]. First, a set of longitudinal Poisson models was constructed to assess the direct influence of individual ultimate and individual distal variables on the rate of multiple risk factor score. We then built a second set of longitudinal Poisson models to assess the direct influence of social ultimate and social distal variables on the rate of multiple risk factor score. A final set of four multivariate models was then constructed to assess the independent longitudinal influence of individual distal/ultimate and social distal/ultimate variables on the rate of multiple risk factor score. Specifically, Model 1 assessed the contribution of individual distal variables; Model 2 assessed the contribution of individual ultimate variables controlling for the effects of individual distal variables; Model 3 assessed the contribution of social distal variables controlling for the effects of individual distal and individual ultimate variables; and Model 4 assessed the contribution of social ultimate variables controlling for the effects of social distal, individual distal, and individual ultimate variables (i.e., all covariates). The log-likelihood ratio statistic was used to assess the contribution of each block of variables to the models [64]. Interaction terms were added to the models to test possible interactions between each covariate and sex as well as between each covariate and time. Sampling and bootstrap weights were used in all analyses to adjust for sample selection and non-response [40]. All statistical analyses were performed using SAS, version 9.1 (SAS Institute Inc., Carry, NC), and SUDAAN, version 9.01 (RTI International, Research Triangle Park, NC).

## Results

### Descriptive findings

Analyses comparing baseline characteristics of children in the study cohort to those of subjects lost during the follow-up or excluded because of incomplete data showed that subjects lost were more often males (*p *= .03), had lower self-esteem (*p *< .001) and greater anxiety (*p *= .02) (Table 1). Subjects lost were also more likely to be from lower socioeconomic status families than children in the study cohort. With respect to lifestyle risk factors, there were no significant differences between the two groups except for ever drinking (*p *= .005) and high body mass index (*p *= .004), which were higher among subjects who were lost to follow-up or excluded from the analysis.

At baseline (2000-2001), 50% of children in the study cohort, aged 10-11 years, were physically inactive, 42% engaged in sedentary behavior, 6% were ever smokers, 6% were ever drinkers and 23% were overweight or obese (Table 2). For males and females, respectively, the prevalence of physical inactivity increased by 18% (*p *< .001) and 15% (*p *= .002), the prevalence of ever smoking increased by 25% (*p *< .001) and 23% (*p *< .001) and the prevalence of ever drinking increased by 41% (*p *< .001) and 43% (*p *< .001) from 2000-2001 to 2004-2005. Overall, females were more physically inactive than males (*p *< .02), while males tended to engage in more sedentary behavior than females especially at the age of 14-15 years (*p *= .002).

**Table 2:** Prevalence of single and multiple behavioral risk factors, by sex, at each time point, National Longitudinal Survey of Children and Youth, 2000-2005

		Time 1^a^		Time 2^a^		Time 3^a^		
		
		(n = 1135)		(n = 1135)		(n = 1135)		*p*
		
		%^b^	95% CI^c^	%^b^	95% CI^c^	%^b^	95% CI^c^	for trend^d^
**Risk factors**								

Physical inactivity^e^	Male	43	36, 49	44	37, 50	61	55, 67	<.001

	Female	57	50, 64	59	52, 65	72	65, 78	.002

Sedentary behavior^f^	Male	49	42, 55	43	37, 50	50	44, 56	.78

	Female	36	30, 42	36	30, 43	36	29, 42	.99

Ever smoking^g^	Male	6	3, 11	12	9, 17	31	26, 38	<.001

	Female	6	3, 10	15	11, 20	29	24, 36	<.001

Ever drinking^h^	Male	7	4, 11	16	12, 22	48	41, 54	<.001

	Female	5	3, 9	14	10, 18	48	41, 55	<.001

High body mass index^i^	Male	24	19, 30	23	17, 30	18	13, 23	.07

	Female	22	17, 29	15	11, 19	14	10, 18	.05

**No. of Risk factors**								

0	Male	31	25, 37	21	17, 26	6	4, 11	<.001

	Female	26	21, 32	22	17, 28	10	6, 16	<.001

1	Male	38	32, 44	40	34, 47	27	22, 32	.003

	Female	44	37, 50	39	33, 47	31	24, 38	.01

2	Male	24	19, 30	27	22, 33	37	31, 43	.002

	Female	24	19, 29	25	18, 32	27	21, 34	.42

3-5	Male	7	5, 11	12	8, 16	30	25, 36	<.001

	Female	6	4, 11	14	10, 19	32	27, 38	<.001

CI = confidence interval.

^a ^Time 1 refers to Cycle 4 (2000-2001), Time 2 refers to Cycle 5 (2002-2003) and Time 3 refers to Cycle 6 (2004-2005) of the National

Longitudinal Survey of Children and Youth.

^b ^Weighted percentage expressed in terms of the proportion of Canadian children aged 10-11 years in Cycle 4 and followed biennially

until Cycle 6 of the National Longitudinal Survey of Children and Youth.

^c ^CIs were computed using bootstrap weights to account for the complex sampling design of the National Longitudinal Survey of Children

and Youth.

^d ^*p *value for linear trend in the percentages of single and multiple behavioral risk factors over time obtained from the polynomial trend test.

^e ^Engaging in organized/unorganized physical activities fewer than 4 times per week.

^f ^Watching television or videos for more than 2 hours per day.

^g ^Ever smoking a cigarette, even a few puffs.

^h ^Ever having a standard drink of alcohol.

^i ^Being overweight/obese, as defined by cutoff points of Cole and colleagues [54].

About 28% of children at baseline aged 10-11 years had none of the five behavioral risk factors, 41% had 1 risk factor, 24% had 2 risk factors and 7% had 3 or more risk factors (Table 2). By the age of 14-15 years, only 8% of these youth had no risk factors, 29% had 1 risk factor, 32% had 2 risk factors and 31% had 3 risk factors or more. There were no significant differences between males and females in the percentage of multiple behavioral risk factors at baseline or across the follow-up period.

### Longitudinal Poisson regression models

In the regression analyses, individual and social variables were grouped into 4 blocks of conceptually-related variables (as per our conceptual framework) to determine their influence on the rate of multiple risk factor score. Longitudinal Poisson models assessing the direct influence of individual distal and individual ultimate variables on the rate of multiple risk factor score showed that both individual distal and individual ultimate variables contributed to the model (Table 3). However, individual distal variables (Table 3, Model 2, Log-likelihood ratio (LLR) = 76.94; degrees of freedom (DF) = 4; *p *< .001) contributed more to the model than individual ultimate variables (Table 3, Model 1, LLR = 35.9; DF = 3; *p *< .001).

**Table 3:** Rate ratios (95% CIs) for the longitudinal associations between selected individual distal and individual ultimate variables and multiple behavioral risk factors (n = 1135), National Longitudinal Survey of Children and Youth, 2000-2005^a^

	Rate ratio^b ^95% CI^c^			Rate ratio^b ^95% CI^c^			Rate ratio^b ^95% CI^c^		
	
	Model 1			Model 2			Model 3		
	
	Individual characteristics			Individual characteristics			Individual characteristics		
	
	Ultimate			Distal			Ultimate		
	Sex			Self-esteem^f^	0.97	0.97, 0.98	Sex		

	Female	1	Referent	Academic performance			Female	1	Referent

	Male	1.01	0.96, 1.06	Poor/very poor	1	Referent	Male	1.00	0.95, 1.05

	Age, years^d^			Average	0.93	0.85, 1.01	Age, years^d^		

	10	1	Referent	Well	0.88	0.80, 0.96	10	1	Referent

	11	1.08	1.02, 1.15	Very well	0.88	0.79, 0.97	11	1.07	1.01, 1.13

	Anxiety^e^	1.02	1.01, 1.03	Time			Anxiety^e^	1.01	1.00, 1.02

	Time			1 (Cycle 4)	1	Referent	**Distal**		

	1 (Cycle 4)	1	Referent	2 (Cycle 5)	1.08	1.03, 1.14	Self-esteem^f^	0.98	0.97, 0.98

	2 (Cycle 5)	1.11	1.06, 1.17	3 (Cycle 6)	1.37	1.30, 1.44	Academic performance		

	3 (Cycle 6)	1.43	1.35, 1.50	Intercept	3.38	3.00, 3.81	Poor/very poor	1	Referent

	Intercept	1.89	1.78, 2.01				Average	0.93	0.85, 1.01

							Well	0.88	0.80, 0.97

							Very well	0.88	0.79, 0.98

							Time		

							1 (Cycle 4)	1	Referent

							2 (Cycle 5)	1.09	1.03, 1.14

							3 (Cycle 6)	1.37	1.30, 1.44

							Intercept	3.09	2.66, 3.60

-2 Log L^g^	1335.42			1294.37			1285.03		

Log L ratio^h^	35.90***			76.94***			86.28***		

DF	3			4			7		

CI = confidence interval; DF = degrees of freedom.

^a ^Multiple behavioral risk factor score was the dependent variable.

^b ^Rate ratios from the multivariate longitudinal Poisson regression model with adjustment for all covariates in the corresponding model and time (cycles).

^c ^CIs were computed using bootstrap weights to account for the complex sampling design of the National Longitudinal Survey of Children and Youth.

^d ^Age at baseline (Cycle 4).

^e ^Anxiety was assessed using a global score ranging from 0 to 14, with higher scores indicating the presence of greater anxiety.

^f ^Self-esteem was assessed using a global score ranging from 0 to 16, with higher scores indicating positive self-esteem.

^g^-2 (log-likelihood) for the model containing each specific block of distal and/or ultimate variables. The -2 (log-likelihood) of the initial (intercept-only + time) model was 1371.31.

^h ^Log-likelihood ratio or change in -2 (log-likelihood) compared to the initial (intercept-only + time) model.

***p < .001.

Analyses assessing the direct influence of social distal and social ultimate variables on the rate of multiple risk factor score also showed that both social distal and social ultimate variables contributed to the model (Table 4). However, social distal variables (Table 4, Model 2; LLR = 254.07; DF = 8; *p *< .001) contributed much more to the model than social ultimate variables (Table 4, Model 1; LLR = 22.03; DF = 5; *p *< .001).

**Table 4:** Rate ratios (95% CIs) for the longitudinal associations between selected social distal and social ultimate variables and multiple behavioral risk factors (n = 1135), National Longitudinal Survey of Children and Youth, 2000-2005^a^

	Rate ratio^b ^95% CI^c^			Rate ratio^b ^95% CI^c^			Rate ratio^b ^95% CI^c^		
	
	Model 1			Model 2			Model 3		
	
	Social characteristics			Social characteristics			Social characteristics		
	**Ultimate**			**Distal**			**Ultimate**		

	Family structure			PMK smoking status			Family structure		

	2 parents	1	Referent	Nonsmoker	1	Referent	2 parents	1	Referent

	1 parent	1.07	0.98, 1.17	Smoker	1.10	1.05, 1.15	1 parent	1.05	0.97, 1.12

	PMK Education			PMK drinking status			PMK Education		

	Low (< 12 years of school)	1	Referent	Nondrinker	1	Referent	Low (< 12 years of school)	1	Referent

	High (≥l2 years of school)	0.94	0.88, 1.01	Drinker	1.01	0.97, 1.06	High (≥l2 years of school)	0.98	0.92, 1.04

	Annual household income			Parent-child relationship^d^	0.99	0.99, 1.00	Annual household income		

	< CAN$30,000	1	Referent	Peer smoking			< CAN$30,000	1	Referent

	CAN$30,000 -59,999	1.01	0.94, 1.09	No peers	1	Referent	CAN$30,000 -59,999	1.01	0.94, 1.08

	CAN$60,000 -89,999	1.07	0.98, 1.16	A few peers	1.14	1.06, 1.22	CAN$60,000 -89,999	1.09	1.00, 1.17

	≥CAN$90,000	1.03	0.93, 1.13	Most/All peers	1.41	1.27, 1.56	≥CAN$90,000	1.04	0.96, 1.14

	Time			Peer drinking			**Distal**		

	1 (Cycle 4)	1	Referent	No peers	1	Referent	PMK smoking status		

	2 (Cycle 5)	1.10	1.05, 1.16	A few peers	1.13	1.06, 1.21	Nonsmoker	1	Referent

	3 (Cycle 6)	1.41	1.34, 1.49	Most/All peers	1.26	1.17, 1.37	Smoker	1.10	1.05, 1.15

	Intercept	2.10	1.94, 2.29	Peer-child relationship^e^	0.99	0.98, 1.00	PMK drinking status		

				Time			Nondrinker	1	Referent

				1 (Cycle 4)	1	Referent	Drinker	1.01	0.97, 1.05

				2 (Cycle 5)	1.06	1.01, 1.11	Parent-child relationship^d^	0.99	0.99, 1.00

				3 (Cycle 6)	1.16	1.09, 1.24	Peer smoking		

				Intercept	2.75	2.45, 3.08	No peers	1	Referent

							A few peers	1.14	1.06, 1.22

							Most/All peers	1.41	1.27, 1.57

							Peer drinking		

							No peers	1	Referent

							A few peers	1.13	1.06, 1.21

							Most/All peers	1.26	1.17, 1.36

							Peer-child relationship^e^	0.99	0.98, 1.00

							Time		

							1 (Cycle 4)	1	Referent

							2 (Cycle 5)	1.05	1.00, 1.11

							3 (Cycle 6)	1.15	1.08, 1.23

							Intercept	2.71	2.39, 3.07

-2 Log L^f^	1349.28			1117.23			1107.07		

Log L ratio^g^	22.03***			254.07***			264.24***		

DF	5			8			13		

CAN = Canadian; CI = confidence interval; DF = degrees of freedom; PMK = person most knowledgeable.

^a ^Multiple behavioral risk factor score was the dependent variable.

^b ^Rate ratios from the multivariate longitudinal Poisson regression model with adjustment for all covariates in the corresponding model and time (cycles).

^c ^CIs were computed using bootstrap weights to account for the complex sampling design of the National Longitudinal Survey of Children and Youth.

^d ^The parent-child relationship was assessed using a global score ranging from 0 to 28, with higher scores indicating a better relationship between parents and child.

^e ^Peer-child relationships were assessed using a global score ranging from 0 to 16, with higher scores indicating a better relationship between the child and his/her peers.

^f ^-2 (log-likelihood) for the model containing each specific block of distal and/or ultimate variables. The -2 (log-likelihood) of the initial (intercept-only + time) model was 1371.31.

^g ^Log-likelihood ratio or change in -2 (log-likelihood) compared to the initial (intercept-only + time) model.

***p < .001.

Adjusted longitudinal Poisson models (i.e., including both individual and social variables) led to similar results as in the models investigating the direct influence of individual distal/ultimate and social distal/ultimate variables. In particular, social distal variables (Table 5, Model 3, LLR = 187.86; DF = 8; *p *< .001), individual distal variables (Table 5, Model 1, LLR = 76.94; DF = 4; *p *< .001), and individual ultimate variables (Table 5, Model 2, LLR = 9.34; DF = 3; *p *< .05) significantly contributed to the rate of multiple risk factor score. Social ultimate variables (Table 5, Model 4, LLR = 10.93; DF = 5; *p = *.05) contributed minimally to the overall rate of occurrence of multiple behavioral risk factors. Among the variables under investigation, PMK smoking (rate ratio (RR) = 1.11; 95% CI = 1.05, 1.16), having reported that a few or most/all of one's peers drank alcohol (a few-RR = 1.12; 95% CI = 1.04, 1.19; most/all-RR = 1.23; 95% CI = 1.14, 1.34) or smoked cigarettes (a few-RR = 1.14; 95% CI = 1.07, 1.22; most/all-RR = 1.41; 95% CI = 1.28, 1.55) were associated with an increased rate of multiple risk factor score (Table 5, Model 4). Higher self-esteem (RR = 0.98; 95% CI = 0.98, 0.99) was related to a decline in the rate of multiple risk factor score (Table 5, Model 4).

**Table 5:** Adjusted rate ratios (95% CIs) for the longitudinal associations between selected individual/social distal and ultimate variables and multiple behavioral risk factors (n = 1135), National Longitudinal Survey of Children and Youth, 2000-2005^a^

	Rate ratio^b ^95% CI^c^			Rate ratio^b ^95% CI^c^			Rate ratio^b ^95% CI^c^			Rate ratio^b ^95% CI^c^		
	
	Model 1			Model 2			Model 3			Model 4		
	
	Individual characteristics			Individual characteristics			Individual characteristics			Individual characteristics		
	
	Distal			Distal			Distal			Distal		
	Self-esteem^d^	0.97	0.97, 0.98	Self-esteem^d^	0.98	0.97, 0.98	Self-esteem^d^	0.98	0.98, 0.99	Self-esteem^d^	0.98	0.98, 0.99

	Academic performance			Academic performance			Academic performance			Academic performance		

	Poor/very poor	1	Referent	Poor/very poor	1	Referent	Poor/very poor	1	Referent	Poor/very poor	1	Referent

	Average	0.93	0.85, 1.01	Average	0.93	0.85, 1.01	Average	0.98	0.88, 1.09	Average	0.98	0.88, 1.09

	Well	0.88	0.80, 0.96	Well	0.88	0.80, 0.97	Well	0.94	0.84, 1.05	Well	0.94	0.84, 1.04

	Very well	0.88	0.79, 0.97	Very well	0.88	0.79, 0.98	Very well	0.96	0.86, 1.08	Very well	0.96	0.85, 1.08

	Time			**Ultimate**			**Ultimate**			**Ultimate**		

	1 (Cycle 4)	1	Referent	Sex			Sex			Sex		

	2 (Cycle 5)	1.08	1.03, 1.14	Female	1	Referent	Female	1	Referent	Female	1	Referent

	3 (Cycle 6)	1.37	1.30, 1.44	Male	1.00	0.95, 1.05	Male	1.01	0.97, 1.05	Male	1.01	0.97, 1.05

	Intercept	3.38	3.00, 3.81	Age, years^e^			Age, years^e^			Age, years^e^		

				10	1	Referent	10	1	Referent	10	1	Referent

				11	1.07	1.01, 1.13	11	1.01	0.96, 1.06	11	1.01	0.96, 1.06

				Anxiety^f^	1.01	1.00, 1.02	Anxiety^f^	1.00	0.99, 1.01	Anxiety^f^	1.01	1.00, 1.01

				Time			**Social characteristics**			**Social characteristics**		

				1 (Cycle 4)	1	Referent	**Distal**			**Distal**		

				2 (Cycle 5)	1.09	1.03, 1.14	PMK smoking status			PMK smoking status		

				3 (Cycle 6)	1.37	1.30, 1.44	Nonsmoker	1	Referent	Nonsmoker	1	Referent

				Intercept	3.09	2.66, 3.60	Smoker	1.10	1.05, 1.16	Smoker	1.11	1.05, 1.16

							PMK drinking status			PMK drinking status		

							Nondrinker	1	Referent	Nondrinker	1	Referent

							Drinker	1.01	0.97, 1.06	Drinker	1.01	0.97, 1.05

							Parent-child relationship^g^	1.00	0.99, 1.00	Parent-child relationship^g^	1.00	0.99, 1.00

							Peer smoking			Peer smoking		

							No peers	1	Referent	No peers	1	Referent

							A few peers	1.13	1.06, 1.21	A few peers	1.14	1.07, 1.22

							Most/All peers	1.40	1.27, 1.54	Most/All peers	1.41	1.28, 1.55

							Peer drinking			Peer drinking		

							No peers	1	Referent	No peers	1	Referent

							A few peers	1.12	1.05, 1.20	A few peers	1.12	1.04, 1.19

							Most/All peers	1.24	1.15, 1.35	Most/All peers	1.23	1.14, 1.34

							Peer-child relationship^h^	1.00	0.99, 1.00	Peer-child relationship^h^	0.99	0.99, 1.00

							Time			**Social characteristics**		

							1 (Cycle 4)	1	Referent	**Ultimate**		

							2 (Cycle 5)	1.05	0.99, 1.10	Family structure		

							3 (Cycle 6)	1.15	1.08, 1.23	2 parents	1	Referent

							Intercept	3.04	2.60, 3.35	1 parent	1.04	0.97, 1.12

										PMK Education		

										Low (< 12 years of school)	1	Referent

										High (≥l2 years of school)	0.99	0.94, 1.05

										Annual household income		

										< CAN$30,000	1	Referent

										CAN$30,000- 59,999	1.00	0.94. 1.08

										CAN$60,000- 89,999	1.09	1.00, 1.17

										≥CAN$90,000	1.04	0.96, 1.12

										Time		

										1 (Cycle 4)	1	Referent

										2 (Cycle 5)	1.04	0.99, 1.10

										3 (Cycle 6)	1.15	1.08, 1.22

										Intercept	2.95	2.49, 3.50

-2 Log L^i^	1294.37			1285.03			1097.17			1086.24		

Log L ratio^j^	76.94***			9.34**			187.86***			10.93*		

DF	4			3			8			5		

CAN = Canadian; CI = confidence interval; DF = degrees of freedom; PMK = person most knowledgeable.

^a ^Multiple behavioral risk factor score was the dependent variable.

^b ^Adjusted rate ratios from the multivariate longitudinal Poisson regression model with adjustment for all covariates in the corresponding model and time (cycles).

^c ^CIs were computed using bootstrap weights to account for the complex sampling design of the National Longitudinal Survey of Children and Youth.

^d ^Self-esteem was assessed using a global score ranging from 0 to 16, with higher scores indicating positive self-esteem.

^e ^Age at baseline (Cycle 4).

^f ^Anxiety was assessed using a global score ranging from 0 to 14, with higher scores indicating the presence of greater anxiety.

^g ^The parent-child relationship was assessed using a global score ranging from 0 to 28, with higher scores indicating a better relationship between parents and child.

^h ^Peer-child relationships were assessed using a global score ranging from 0 to 16, with higher scores indicating a better relationship between the child and his/her peers.

^i ^-2 (log-likelihood) for the model containing each specific block of distal and ultimate variables. The -2 (log-likelihood) of the initial (intercept-only + time) model was 1371.31.

^j ^Log-likelihood ratio or change in -2 (log-likelihood) is presented for each block of distal or ultimate variables entered in the multivariate model. At each step, the log-likelihood of the bigger model was compared to the log-likelihood of the previous smaller model.

*p = .05; **p < .05; ***p < .001.

## Discussion

This study assessed the longitudinal influence of selected conceptually-related individual and social variables on the rate of occurrence of multiple behavioral risk factors in a representative cohort of Canadian youth. Our results first indicate a 23% increase in the percentage of youth with 3 or more risk factors and a 20% decline in the percentage of youth with 0 risk factors across the follow-up period. As expected, both distal and ultimate variables contributed to the likelihood of the occurrence of multiple behavioral risk factors during follow-up. However, contrary to our expectation, the log-likelihood ratio statistic indicated that distal variables, particularly social distal factors, contributed more to the longitudinal Poisson model than ultimate variables. This finding is important because distal variables tend to be actually easier to modify through effective interventions compared to ultimate variables [39]. We are aware of no other study assessing the influence of blocks of distal or ultimate variables on the rate of occurrence of multiple behavioral risk factors in either youth or adults. Hence, it is difficult to compare results of this study with other relevant reports. Nevertheless, our results corroborate findings of a recent cross-sectional study, also based on the Theory of Triadic Influence, where friends' substance use, a social distal variable, was found to be associated with both alcohol use and cigarette smoking in two convenience samples of Russian and American high school students in grade 10. In contrast, depression, an individual ultimate variable, was not associated with either behavior in the same study [65].

Of the social distal variables considered in our study, caregiver smoking was linked to an 11% increase in the rate of multiple risk factor score among youth. Adverse parental health behaviors have been associated with unhealthy behaviors of their children in only two cross-sectional studies of multiple behavioral risk factors for chronic diseases [25,27], as well as longitudinal studies of single risk factors including cigarette smoking [66] and obesity [67,68]. Two other social distal variables including having peers who smoked cigarettes and having peers who drank alcohol increased the likelihood of having multiple risk factors by up to 41% and 23%, respectively. These findings are consistent with results of other observational studies where peer smoking [69] and peer drinking [70] were associated with the occurrence of single behavioral risk factors among adolescents. However, we are aware of no other longitudinal study investigating the potential association between peer unhealthy lifestyles and the rate of occurrence of multiple behavioral risk factors for chronic diseases among youth. Nevertheless, as suggested by several social bonding theories, parents and peers are perceived as role models, and are thought to affect youth health behaviors by shaping perceived social norms to adopt or maintain health behaviors [39]. Moreover, these results concord well with findings of existing intervention literature where effective interventions have tended to focus on social distal factors (such as parental and peer behaviors and parental involvement) for preventing risk factors such as cigarette smoking, unhealthy diet consumption, and obesity [71,72]. Hence, these findings suggest the importance of interventions in the child's immediate social environment to support multiple-behavior change.

Among the individual distal variables studied, higher self-esteem was associated with a decline in the rate of multiple risk factor score among youth. This finding is concordant with results of a longitudinal study where lower self-esteem was linked to single health-compromising behaviors including cigarette smoking, alcohol use and problem behavior among adolescents aged 15 years [73]. It has been suggested that individuals with stronger self-esteem tend to place greater value on self-determination and possess a strong will to modify, regulate or restrain their health behaviors [39].

The social ultimate variables considered in this study (i.e., parental education, household income and family structure) contributed minimally to the overall rate of multiple risk factor score. The evidence from the literature regarding the association of socioeconomic status and multiple behavioral risk factors for chronic diseases has been mixed. For example, in a recent cross-sectional study, family structure and education, but not income, were associated with multiple chronic disease behavioral risk factors among Canadian youth aged 10-15 years [27]. An Australian study found a cross-sectional association between family income and the co-occurrence of behavioral risk factors among adolescents aged 14 years [21], while two American cross-sectional studies did not find an association between parental level of education and the presence of multiple behavioral risk factors in children and adolescents aged 11 to 15 years [25,26]. These divergent findings may be partly attributed to the use of different definitions for parental education and household income across these cross-sectional studies. Hence, there is a need for additional research on the association of family socioeconomic status and the occurrence of multiple chronic disease behavioral risk factors among youth, particularly using longitudinal designs.

This study comprised some limitations. First, adopting a theory-based approach has its drawbacks as it often relates poorly to the real world. For example, the ultimate tier of influence may comprise additional factors (such as factors related to the broader socioeconomic context of youth) not included in the study. Also, the mechanisms by which distal and ultimate factors influence multiple behaviors may be more complex than what is depicted in the conceptual framework of the study. Nevertheless, the Theory of Triadic Influence does recognize that there are possible interstream pathways between different levels of influence. For example, the child's age (an individual ultimate variable) might have its primary influence on the child's sense of self (an individual distal variable) but it might also, to a lesser degree, influence how well the child bonds with others (a social distal variable) [39].

Second, some selection bias may have occurred due to loss to follow-up or the exclusion of subjects because of incomplete data. In particular, the sample may have been somewhat selected towards youth from more affluent and healthy families. Since single and multiple behavioral risk factors tend to be more prevalent among youth of low socioeconomic status [23], the observed associations herein may be even stronger in reality because of the limited inclusion of youth from less affluent families. Also, although our final multivariate model adjusted for all covariates, it remains possible that additional unaccounted factors explain our findings.

Health behaviors were self-reported in the NLSCY and thus subject to recall and social desirability biases. Moreover, the measure of body mass index was based on parent-reported height and weight for children aged 10-11 years, and self-reported height and weight for adolescents aged 12 years or over in the NLSCY. It has been suggested that when parents report their children's height and weight, overweight and obesity may be overestimated, mainly because parents tend to underestimate their children's height [74]. In contrast, self-reported height and weight tend to yield slightly lower estimates of body mass index compared to objective measures [74].

Lastly, the five behavioral risk factors under study were summed to create a multiple risk factor score. To construct this score, each behavior was dichotomized, the practice of which necessarily entails some loss of information. However, since behaviors under study were measured on different scales, dichotomization according to national/international cutoff points was judged appropriate. Also, some authors have questioned the use of additive indices where risk factors are attributed equal weights [75,76]. In contrast, other experts have shown that use of equally weighted risk factor indices results in the identification of very similar at risk populations than those identified by unequally weighted risk factor indices [77,78].

Despite these limitations, this study had several important strengths including its use of a nationally representative sample of youth, the use of an integrative theoretical framework to guide the study of determinants of multiple behavioral risk factors, and its longitudinal design.

## Conclusions

This study contributed new knowledge about determinants of multiple chronic disease behavioral risk factors among youth. In particular, this longitudinal investigation showed that individual distal and social distal variables exerted a stronger influence on the rate of co-occurrence of behavioral risk factors among youth, compared to individual/social ultimate variables. Specifically, parental and peer unhealthy lifestyles were associated with an elevated rate of multiple risk factor score. Youth with stronger sense of self over time were less likely to have multiple behavioral risk factors. These results support the use of distal variables as potential targets in public health interventions aiming to curb the increased rate of occurrence of multiple behavioral risk factors among youth. Further research is needed to evaluate the influence of ultimate variables, often considered the root causes of behaviors and hard to modify [39], on multiple behavioral risk factors among youth.

## Competing interests

The authors declare that they have no competing interests.

## Authors' contributions

AA designed the study, conducted the analyses, interpreted the data and wrote the manuscript. GP contributed to data interpretation, provided comments on and reviewed the manuscript critically for important intellectual content and quality. Both authors have read and approved the final manuscript.

## Pre-publication history

The pre-publication history for this paper can be accessed here:

http://www.biomedcentral.com/1471-2458/12/224/prepub
